# Admixture Mapping Provides Evidence of Association of the VNN1 Gene with Hypertension

**DOI:** 10.1371/journal.pone.0001244

**Published:** 2007-11-28

**Authors:** Xiaofeng Zhu, Richard S. Cooper

**Affiliations:** 1 Department of Epidemiology and Biostatistics, School of Medicine, Case Western Reserve University, Cleveland, Ohio, United States of America; 2 Department of Preventive Medicine and Epidemiology, Stritch School of Medicine, Loyola University, Maywood, Illinois, United States of America; North Carolina State University, United States of America

## Abstract

Migration patterns in modern societies have created the opportunity to use population admixture as a strategy to identify susceptibility genes. To implement this strategy, we genotyped a highly informative ancestry marker panel of 2270 single nucleotide polymorphisms in a random population sample of African Americans (N = 1743), European Americans (N = 1000) and Mexican Americans (N = 581). We then examined the evidence for over-transmission of specific loci to cases from one of the two ancestral populations. Hypertension cases and controls were defined based on standard clinical criteria. Both case-only and case-control analyses were performed among African Americans. With the genome-wide markers we replicated the findings identified in our previous admixture mapping study on chromosomes 6 and 21 [1]. For case-control analysis we then genotyped 51 missense SNPs in 36 genes spaced across an 18.3 Mb region. Further analyses demonstrated that the missense SNP rs2272996 (or N131S) in the VNN1 gene was significantly associated with hypertension in African Americans and the association was replicated in Mexican Americans; a non-significant opposite association was observed in European Americans. This SNP also accounted for most of the evidence observed in the admixture analysis on chromosome 6. Despite these encouraging results, susceptibility loci for hypertension have been exceptionally difficult to localize and confirmation by independent studies will be necessary to establish these findings.

## Introduction

Hypertension is a consequence of common lifestyle patterns in modern society and makes an important contribution to risk of cardiovascular disease. The prevalence of hypertension varies among ethnic populations in the US from 25 to 40% and is an attributable cause for approximately 13% of deaths in whites and 24% in blacks [2]. Blood pressure is a moderately heritable trait and results from the combined effect of a complex set of genetic and environmental influences, with genes cumulatively accounting for 30% of the population variance [3]. Genome-wide linkage analysis has been widely applied in efforts to identify genomic regions harboring genes affecting the risk of hypertension. A recent review of 20 genome scans suggested that a large number genes, each exerting a small effect, is the most likely molecular architecture underlying hypertension [4]. The observed effects are highly inconsistent, however, and it is well recognized that linkage analysis has limited power when applied to complex traits [5]; locus heterogeneity may further contribute to this observed inconsistency.

As an alternative to linkage methods, numerous candidate genes selected on physiological and/or metabolic criteria have been examined using single nucleotide polymorphisms (SNPs) or associated haplotypes. Despite the myriad of reports, meta-analyses have identified few candidate loci with consistent effects across population samples [6]. For example, the genes underlying physiologic systems that control BP, like the renin-angiotensin axis, have been extensively studied, and yielding inconsistent results [7], [8]. Genome-wide association studies based on 100,000 or more SNPs are now technically feasible [9], [10] and initial results with macular degeneration, obesity, type 1 and type 2 diabetes, prostate cancer and multiple sclerosis, among others, suggest promise for this method [11]–[19]. These studies are still quite expensive, however, and can only be conducted by a limited number of laboratories.

On the basis of recent theoretical work it has been suggested that the information generated by recent admixture of historically separated populations can help to map disease-associated genes [20]–[29]. Admixture mapping can be more powerful than traditional linkage analysis when the relative risk in the parental populations is substantially different and much less genotyping is required in comparison to association analysis based on linkage disequilibrium. Admixture mapping may also be less sensitive to genetic heterogeneity [30]. Based on these theoretical propositions, Zhu et al. performed the first large-scale genome wide admixture mapping study in African Americans using the markers designed for a family-based linkage study [1]. In these analyses the distribution of marker location-specific ancestry was shifted upward in hypertensive cases versus normotensives and this shift was largely due to the loci on 6q24 and 21q21, indicating that genetic variants in these two regions may influence the risk of hypertension. Since the marker information content for inferring ancestry was relatively low in this initial study, the findings require further confirmation. Consequently using a large, multi-ethnic population sample, we conducted an admixture mapping study by genotyping a marker panel that is highly informative for ancestry for the African-American population [31]. In this paper we describe the admixture linkage results for hypertension in the Dallas Heart Study, followed by an association study of all missense SNPs in the region identified by the admixture mapping.

## Results

The demographic and descriptive characteristics of the hypertensive cases and controls are presented in Table 1 for the European-American, African-American and Mexican-American population samples. Cases were on average older than controls in all three groups, as anticipated; the percentage of treated cases was similar.

**Table 1:** Descriptive characteristics of the study subjects from each of racial/ethnic groups (means±sd)

	Control			Case		
	Black	White	Mexican American	Black	White	Mexican American
Number (male/fem)	880 (389/491)	662 (316/346)	453 (192/261)	863 (347/516)	338 (162/176)	128 (53/75)
Age	40.8±9.3	43.3±9.7	38.7±8.1	48.8±9.4	47.9±9.9	45.9±10.1
BMI	29.2±7.2	27.1±5.9	28.9±5.6	31.7±7.7	30.8±7.0	32.1±7.1
SBP	119.7±13.4	117.9±12.3	116.6±11.3	143.1±24.8	136.7±17.2	138.5±19.8
DBP	75.5±9.0	74.7±8.0	73.8±7.5	86.4±14.0	84.6±11.0	83.9±9.6
No. Treated	0	0	0	663 (76.8%)	255(75.4%)	94(73.4)

### Admixture mapping in African-Americans

**Figure 1:**
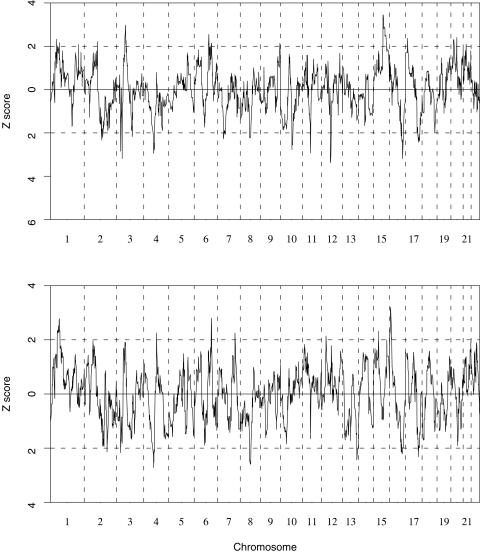
The genome-wide Z-scores. Top: the Z-scores calculated using hypertensive cases only ; Bottom: the Z-scores calculated based on case-control samples.

2,270 ancestry informative SNPs located on 22 autosomes selected from the SNP panel of Smith et al.[31] were successfully genotyped. After examination of Hardy-Weinberg equilibrium (HWE) for possible genotyping errors and background linkage disequilibrium which may violate the assumption of the method [25], only 1,890 SNPs were used in further analyses. Our analysis used the software ADMIXPROGRAM, which is based on the hidden Markov model using the continuous gene flow model [25]. The estimated number of generations since the occurrence of population admixture was 12 in this African-American sample. We estimated each individual's average African and European ancestries (Table S1)(European ancestry = 15.8±7.6% in cases, and 16.9±8.3% in controls) as well as ancestry at each marker locus in cases and controls separately (Table S2). We then calculated the genome-wide case-only and case-control Z scores, as presented in figure 1. The Z score can be used to evaluate evidence for a disease variant at each location and it asymptotically follows the standard normal distribution [21], [25]. We observed 6 markers with both |Z_case_| and |Z_case-control_|>2.0 distributed on 6 chromosomes (Table 2). Among them, the signal identified by SNP rs703193 (137cM) on chromosome 6 falls within the region identified in our previous study [1]. To further rule out that the possibility that the evidence observed on chromosome 6 results from the bias resulting either from mis-estimation of a specific marker allele's frequency in ancestral populations or background linkage disequilibrium, we calculated both the case-only and case-control Z scores using different sets of SNPs as suggested by Reich et al. [32] (Table 3). Similar evidence was observed at rs703193 when we used the SNPs with an adjacent distance greater than 1 cM. The evidence remains when either odd SNPs or even SNPs were used. Gender-specific analysis suggested that women contribute more evidence, which may in part reflect the inclusion of more hypertensive women in the study (Table 1). The relative risk of hypertension due to two copies of African ancestry allele in the region around SNP rs703193 was 1.32 (95% CI: (1.04, 1.67), p = 0.023). We also examined the region on chromosome 21 identified in our previous study [1]. Interestingly, the admixture mapping evidence in the region around rs380417 on chromosome 21 was weaker than on chromosome 6q, but with a stronger relative risk of African ancestry at rs380417 = 1.48 (viz, 95%CI: (1.15, 1.89), p = 0.0021).

**Table 2:** Marker locations with the maximum absolute Z score larger than 2.0 for association with hypertension

Marker	Location (cM)	Excess of African Ancestry in cases	African-American Z_case_	African-American Z_case-control_	Genome-wide p-value a
Rs428854	1p: 73.7	0.018	2.2	2.8	0.58
Rs184008	4q:81.8	−0.024	−2.9	−2.2	0.49
Rs703193	6q:137	0.018	2.1	2.8	0.18^b^
rs784037	8q:74.8	−0.018	−2.1	−2.5	0.80
Cv490881	16q:106	−0.026	−3.2	−2.0	0.34
Cv118741	17q:105	−0.020	−2.4	−2.3	0.80
Rs380417	21q: 26	0.017	2.1	1.4	0.66^b^

a The genome-wide p-value was calculated by the number of times with |Z_case_|, |Z_case-control_| in African-Americans greater than the corresponding observed Z scores among the 1000 simulations.

b The p value was calculated using one-side test.

**Table 3:** Zcase and Zcase-control scores at rs703193 when different set of ancestral informative SNPs are used.

	Excess of African Ancestry (%)	Z_case_	Z_case-control_
**All SNPs (1890 SNPs)**	1.8	2.1	2.8
**SNPs>1cM (1236 SNPs)**	1.8	2.8	2.6
**Odd SNPs**	1.9	2.4	3.0
**Even SNPs**	1.1	1.7	2.1
Female, all SNPs	3.2	3.1	2.3
Male, all SNPs	1.5	1.3	1.1

We next empirically explored the probability of Z_case_ and Z_case-control_ scores greater than we observed on chromosome 6 and 21 in the African-American sample when no genetic variants contributed to a trait. We applied a one-sided test for the African-American sample because of previous evidence in this region. We then simulated 1,000 data sets for both samples with the estimated parental allele frequencies and 12 generations based on a continuous gene flow model [21]. The numbers of analyses with Z_case_>2.1, Z_case-control_>2.8 for chromosome 6, and Z_case_>2.1, Z_case-control_>1.4 for chromosome 21, were summarized. Among 1,000 simulations, we observed 184 instances where both Z_case_>2.1, Z_case-control_>2.8 and 688 instances where both Z_case_>2.1 and Z_case-control_>1.4 respectively, indicating that our observations on both 6q and 21q do not reach genome-wide significance. However, when we restricted the analysis to the regions between 129–166 cM on chromosome 6 and between 0–30 cM on chromosome 21 identified in our previous study [1], we only observed 4 and 11 instances respectively (p = 0.004 and 0.011), suggesting that our results do in fact replicate the previous findings. We performed similar analyses with the other 5 regions, using a two-side test since no prior evidence was observed in these regions, and none reached genome-wide significance (Table 2).

We performed similar analysis using the software STRUCTURE. Among the African Americans, European ancestry was estimated as 15.8%±8.3% in cases, and 17.0±9.1% in controls, which are the same as we obtained with ADMIXPROGRAM. The detailed European ancestry for each individual estimated by both methods is presented in Supplement Table S1. The correlation between the two methods is 99.96%. We also calculated the case-only and case-control Z scores by excluding the estimates of an individual's marker-location specific ancestry if the difference of the estimates between the two methods is greater than 0.1. No substantial changes in the Z scores were observed (Table S2).

### Association analysis on chromosome 6

**Figure 2:**
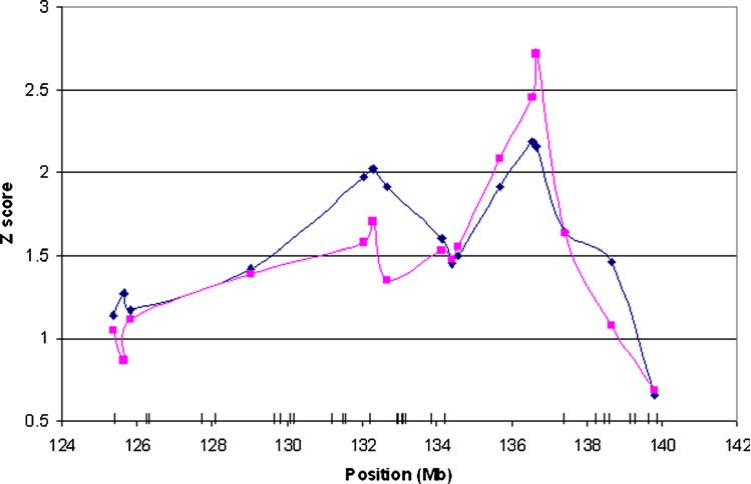
The Z-scores and 39 missense SNPs on chromosome 6. The region represents 18.3 Mb with Z-score >1,0. (in black): case-only Z score (in red) : case-control Z score; small vertical bars on the X-axis represent the location of the 39 missense SNPs.

We next focused on the region around rs703193 defined by Z_case_>1.0 on chromosome 6 which encompassed 18.3 Mb and 36 genes. We hypothesized that missense SNPs are more likely responsible for the evidence identified by admixture mapping [5], [33]. We identified 51 missense SNPs in the 36 genes from public databases and typed them in the African-American sample (Figure 2). Eight of these SNPs were found to have a MAF<5% and an additional 4 were in strong HWD (p<0.01). Admixture mapping has extremely limited power to identify rare variants so these 12 SNPs were excluded from further analyses. We performed logistic regression on each of the remaining 39 SNPs assuming an additive model with adjustment for gender, age, BMI and estimated individual ancestry (IA) based on the original 1890 marker panel. We chose to focus only on the additive model in this step because the reduction of multiple comparisons more than compensates for the small sacrifice in power associated with this strategy.

**Figure 3:**
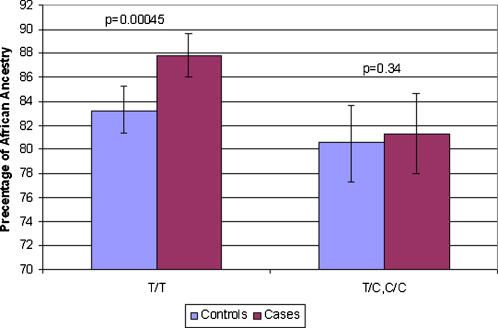
African ancestry in cases and controls grouped by genotypes of SNP rs2272996. A significant excess of African ancestry between cases and controls who were TT was observed but among those that bore the genotype TC and CC.

We also adjusted for IA to reduce the likelihood of false association that results from population stratification. Association with hypertension was identified at α = 0.05 significance level at rs2244008 (A/G) and rs2272996(C/T) in the LAMA2 and VNN1 genes, respectively (Table 4). We then examined whether these two SNPs captured the evidence from admixture mapping. First, we attempted to isolate the ancestry effect. After inferring each individual's ancestry at the admixture peak (137cM) we repeated the logistic regression analysis in the subgroup whose African ancestry was >80% at this locus, assuming that this reduced any potential effects of population stratification. While the result for rs2244008 was unchanged, the association of rs2272996 with hypertension was strengthened (p = 0.00248) despite the reduction in sample size. Based on a permutation test this SNP remained significant (p = 0.045) after adjustment for multiple comparisons (See Methods). SNP rs2272996 is located 3.6 Mb from rs703193, with virtually no linkage disequilibrium between these two SNPs (D' = 0.008, 95% CI (−0.01–0.12)). To determine if two SNPs with this relationship can potentially be in LD in an admixed population similar to African-Americans, we calculated the LD between these two SNPs in a synthetic admixed population using HapMap YRI and CEU date. We assumed that the admixture proportions were 84% African and 16% European. Under these conditions D' was estimated as 0.095, which is within the 95% CI of the finding in our data, suggesting that an AIM used for admixture mapping is not necessarily in strong LD with a SNP associated with the phenotype. We also performed logistic regression for SNP rs703193 adjusting for gender, age, BMI and IA and no evidence of association was found. To further evaluate the plausibility of the evidence for rs2244008 we compared the allele frequency between YRI and CEU samples from HapMap data [10], as well as in our own European-American sample. The frequencies were almost the same, confirming that this SNP is unlikely to account for the evidence observed by the admixture mapping. In contrast, for rs2272996 the allele frequencies were substantially different between YRI and CEU and between YRI and our own European-American sample (Table 4). Further analysis suggested that applying a recessive model rs2272996 (TT vs TC and CC) yielded the most significant association, with hypertension risk increased in the presence of the TT genotype (p = 0.0021, Table 5). Further correction for multiple comparisons of 39 SNPs and additive and recessive modes of inheritance yielded a p-value of 0.06 for rs2272996. We therefore hypothesized that the excess of African ancestry at the peak location (137cM) in cases was accounted for by those who carried the TT genotype. Figure 3 presents African ancestry at the peak location for people who carry TT compared to the remaining genotypes between cases and controls. As expected, we observed a significant excess of African ancestry between cases and controls who were TT (p = 0.00045) but not those that were TC and CC (p = 0.34). These results suggest that rs2272996 is a potential candidate to explain the admixture mapping evidence. When we added rs2272996 in an admixture mapping analysis and the Z score at rs227299 did not improve. This result is reasonable because the information content after adding rs2272996 does not increase, given the AIMs used in this region.

**Table 4:** Two SNPs identified by logistic regression analysis assuming additive model at significance level 0.05. Gender, Age, age^2^, BMI and African ancestry were adjusted. Minor allele was coded as the risk allele.

							Adjust for ancestry		African Ancestry>80%	
SNPs	Genes	MAF (AA)	EA	HA	YRI HapMap	CEU HapMap	Odds ratio (95% CI)	P-value	Odds ratio (95% CI)	P-value
**Rs2244008(A/G) (T2636A)**	LAMA2	11.4(G)	7.8	7.3	7.5	5.8	0.76(0.6, 0.96)	0.023	0.72(0.54,0.95)	0.021
**Rs2272996(C/T) (N131S)**	VNN1	18.0(C)	27.6	18.4	18.3	34.2	0.81(0.67,0.95)	0.035	0.68(0.53,0.87)	0.00248

**Table 5:** Analysis of rs2272996 in African Americans, Mexican Americans and European Americans using recessive model (TT vs TC, CC). Gender, Age, age^2^, BMI and African ancestry were adjusted. Allele T was coded as the risk allele.

	Odds ratio (95% CI)	P-value	Freq of T allele	
			cases	controls
**African Americans**				
** Adjusting for ancestry (all people included)**	1.29(1.03,1.61)	0.0296	0.832	0.808
** People with African ancestry>80%**	1.57(1.18,2.09)	0.0021	0.840	0.804
**Mexican Americans**	1.82 (1.09, 3.02)	0.021	0.856	0.805
**European Americans**	0.79 (0.60, 1.04)	0.089	0.696	0.739

We then analyzed rs2272996 by assuming a recessive model in both European Americans and Mexican Americans using logistic regression with adjustment for gender, age, age^2^, and BMI. We observed significant evidence of association between rs2272996 and hypertension in Mexican Americans (p = 0.021), where the TT genotype increased the risk of hypertension (Table 5). Among European Americans, on the other hand, a similar analysis demonstrated the opposite–albeit non-significant–association (TT genotype protective, odds ratio = 0.79 (p = 0.089). This result is consistent with the findings in African Americans, where the association increased when analysis was limited to individuals with >80% African ancestry at the peak by admixture mapping. We then estimated the population attributable risk of rs2272996 in the African-American and Mexican populations. Under the recessive model we obtained a population attributable risk of 16% in African-Americans, and 35% in Mexican-Americans; this result occurs because T-the risk allele–is relatively common in both populations.

## Discussion

Admixture mapping has been proposed as an alternative to traditional linkage and association studies and in theory holds great promise for selected traits [20]–[29]. In the first application of this strategy to mapping complex traits, Zhu et al. performed a genome-wide admixture mapping of hypertension in an African-American sample based on a set of microsatellite markers designed for traditional linkage analysis and identified two regions on chromosome 6 and 21 that potentially harbored hypertension susceptibility genes [1]. Reich et al. [32] also reported a locus on chromosome 1 associated with multiple sclerosis by admixture mapping. More recently, linkage analysis followed by fine mapping identified a chromosome region harboring genes for susceptibility to prostate cancer [34] and this locus was subsequently replicated with admixture mapping [35]. In this report we present additional evidence from genome-wide admixture mapping for hypertension in 1743 unrelated African Americans using a highly informative SNP panel [31]. This panel extracted on average 89% of the information of ancestry (measured by SIC) [25] for the African-American sample and as a consequence the results from both case-only and case-control tests were reasonably robust. Our estimate of the average information for ancestry is higher than was found in the original report for this SNP panel [31]. This may be explained by the fact that we assumed a 50%–50% mixture when no SNPs are genotyped while Smith et al. assumed a 79%–21% mixture. Under the latter assumption the estimated average information was 65%, slightly less than the 71% suggested by Smith et al. [31]. The estimated ancestral allele frequencies in the parental populations are close to the corresponding observed frequencies in contemporary African and European populations, suggesting that our model fits the data well. We observed 6 regions with consistent excess of African or European ancestry in both case-only and case-control analyses, including the region on chromosome 6 identified in our previous study [1]. However, simulation studies indicate that we did not find any region that reaches genome-wide significance in this study. Although the regions on chromosome 6 and 21 identified in our previous study are large (37cM and 30 cM, respectively), our simulations indeed suggest that the present result is unlikely to be due to chance. It is interesting that we observed stronger evidence but less significant association with the African-derived allele on chromosome 6 than on chromosome 21. Several reasons may explain this outcome. 1) The power of admixture mapping is dependent on the underlying genetic model, with the recessive mode of inheritance being more powerful than additive or dominant modes [21]. A recessive effect for the T allele of missense SNP rs2272996 in VNN1 gene best explains the evidence, suggesting it is reasonable to observe better admixture mapping evidence on chromosome 6 than 21. 2) The power of admixture mapping is dependent on the disease allele frequency difference between two ancestral populations, while the power of association is dependent on the linkage disequilibrium between the underlying disease variant and the test marker, which is the ancestry in this case, and the disease allele frequency in the admixed population. We performed a power analysis by assuming that the missense SNP rs2272996 is the true causative variant. We further assumed that the odds ratio of the TT genotype vs others in African ancestry population is 1.57, as estimated in Table 5. Since we failed to observe evidence of association in the European American population, the odds ratio was simply placed at 1.0, which leads to the estimate of the relative population risk ratio of 1.38. Under these assumptions, our power analysis suggests that, post hoc, we had 38% power to detect a region reaching the genome wide significance with the sample size in the current study. Thus, our study clearly has relatively low power to find similar locus. 3) A spurious finding is still possible because of the statistical fluctuation despite our previous report. Further association analysis is necessary to rule out the possibility that this is a false positive finding.

We performed our analysis based on a method which directly maximizes the likelihood function from the hidden Markov Model using the EM algorithm, allowing for uncertainty in model parameters, such as the allele frequencies in the parental populations [25]. Simulations suggested that this method can perform as well as the widely used Bayesian MCMC method STRUCTURE for the data generated from various population admixture models.[25] However, we believe the results can be regarded with greater confidence if different approaches lead to similar results, as suggested by others [36]. We thus performed similar analysis using STRUCTURE, although we are also aware that other similar MCMC based software has become available [22]–[24]. The difference between ADMIXPROGRAM and STRUCTURE includes the approach to estimation of a large number of parameters, eg, ADMIXPROGRAM directly maximizing the likelihood function based on EM algorithm while STRUCTURE uses a Gibbs sampling scheme. The other difference involves the transition probability in the Hidden Markov model. ADMIXPROGRAM uses the transition probability derived from a continuous-gene-flow model and STRUCTURE uses linkage model by assuming that chunks of chromosomes are derived from ancestral populations and the breakpoints between successive chunks occur randomly [25], [37]. Despite the substantial difference of the two approaches, the results are almost identical, providing further confidence that our results are not biased of the selection of the statistical method.

Several publications [22], [23], [25], [26], [38] have demonstrated that admixture mapping can be seriously biased by background LD between adjacent SNPs when this phenomenon is not properly considered. We thus examined the background LD in European Americans and dropped the SNPs that are in strong LD. Since the maker panel was initially selected to minimize background LD, eliminating additional selected markers based on this characteristic had a very limited effect. We further repeated the analyses using different sets of SNPs: (eg, odd or even SNPs , those with adjacent distance>1 cM) and the results on chromosome 6 were essentially the same. We note, however, that more information might have been obtained by keeping those SNPs that were in LD when using the Markov Hidden Markov Model method recently proposed by Tang et al.[26].

We next followed up the region on chromosome 6 by genotyping 51 missense SNPs in 36 genes spaced across the 18.3 Mb region. Searching for disease variants by testing all functional SNPs has been advocated by Risch [5] and Risch and Botstein [33] and this strategy successfully identified the sixth type 1 diabetes locus by examining all functional SNPs across the genome [13]. We adapted this strategy in the chromosome 6 region and identified a missense SNP rs2272996 (or N131S) in the VNN1 gene significantly associated with hypertension in African Americans after adjusting for multiple comparisons; this association was replicated in Mexican Americans. A non-significant association in the opposite direction was observed in European Americans. (No correction was made for multiple comparisons in Mexican Americans and European Americans since we tested only rs2272996 in these two populations.) Further analysis also indicates that this SNP accounts for most of the evidence of excess of African ancestry observed in this region for African Americans. Thus, the association of SNP rs2272996 to hypertension is unlikely to be due to chance alone, although additional replication studies in independent studies are necessary. It should also be noted that our search of causative variants was not comprehensive and other susceptibility variants may well exist in this region.

We were also puzzled by the direction of the risk estimate associated with allele T in different populations. Theory suggests that the divergent risk relationship at this locus in ancestral populations will increase the power of admixture mapping method. However, the different patterns of risk in these populations cannot be explained with the data currently available, but could reflect gene-gene or gene-environmental interactions [39]. It is possible that this locus was under different selective pressure in different populations, however, the evolutionary processes that have molded susceptibility to chronic cardiovascular disease have not been defined. Whether the at-risk alleles arose under positive selection or a neutral-equilibrium model therefore cannot be determined. The suggestion has been made that susceptibility variants for hypertension may have been under selection pressure due to the climate adaptation, however most of the loci identified under this assumption have not been replicated in association analyses [40]–[42]. Under this model, the variants are unlikely to be deleterious and could be common. Voight et al.[43] proposed a method to detect signals of very recent positive selection in the human genome using HapMap data and the VNN1 gene is located in one of the regions with strong positive selection pressure in YRI sample. VNN1 belongs to the vanin family of proteins, including secreted and membrane-associated proteins which have been reported to participate in hematopoietic cell trafficking and to possess pantetheinase activity which may play a role in oxidative-stress response [44]. It has been recently suggested that increased oxidative stress may antedate hypertension and contribute to its pathogenesis [45]. More recently, VNN1 has been suggested as a novel gene for cardiovascular disease risk, strongly associated with expression levels of several lipid metabolism/CVD-risk genes, [46], [47] although the underlying pathway of VNN1 and other CVD-risk genes remains unknown.

In summary, we conducted a genome-wide search for susceptibility loci for hypertension using admixture mapping in African Americans. Further association studies identified a missense SNP rs2272996 in the VNN1 gene that may explain the evidence identified through admixture analysis. While requiring further confirmation, this finding demonstrates the potential for the admixture approach and suggests it could be a tool for use in studies to define genes affecting selected complex traits. In addition, our study only surveyed the non-coding SNPs on the region on chromosome 6. A more comprehensive assessment of variants in this region will be required to provide assurance that the causative variants associated with hypertension have been identified.

## Materials and Methods

### Samples

The design and methodology of the Dallas Heart Study have been reported elsewhere [48]. In brief, a multistep probability sample of civilian, non-institutionalized English- or Spanish-speaking Dallas County residents were recruited from households. African Americans were oversampled to ensure that they represented 50% of the final cohort. Eligible subjects were invited to participate in three stages of the project including two home visits during which a survey was administered and blood and urine specimens were obtained and a third visit at the University of Texas at Southwestern Medical Center during which imaging studies were accomplished. Three thousand three hundred and ninety eight individuals (52% black) participated in the blood drawing that led to DNA isolation. Informed consent was obtained from all participants and the Institutional Review Board of the University of Texas Southwestern Medical Center approved the study protocol.

Race-ethnicity was based on self-identification. Subjects were asked in separate questions “Are you of Mexican American origin” and “What is your primary racial or ethnic identity.” The following options were provided for the latter question: Black/African American; White/Caucasian; American Indian, Alaska Native; Asian, Pacific Islander, East Indian; Other (Specify). Self-identified whites were used as a proxy for the European founding populations in the genetic analyses.

### Phenotype

#### Blood pressure/hypertension

At each of the three visits (home visits 1 and 2 and visit 3), 5 sets of blood pressure measurements were obtained using an automatic oscillometric device (Welch Allyn, Series #52,000, Arden, NC) with an appropriately sized blood pressure cuff. This device has been validated against catheter measurement of arterial pressure [48]. The BP for analysis was considered the average of measurements 3 through 5 at each visit (total 9 readings). Hypertensive cases were defined as persons with either a systolic BP> = 140 or diastolic BP> = 90, or current treatment with an antihypertensive medication.

### Genotyping

For each subject, DNA sample was individually genotyped by *Perlegen Sciences* for the complete marker set defined by Smith et al [31]. Among them, 2,270 SNPs were successfully genotyped. Fifty one additional missense SNPs in 36 genes on chromosome 6 were identified from National Center for Biotechnology Information (NCBI) build 34, and then genotyped by Perlegen Sciences.

### Selection of admixture marker set

Hardy-Weinberg equilibrium (HWE) was first examined for all the 2,270 successfully genotyped SNPs on 22 autosomes in the three populations separately using the software Haploview [49]. Thirty two SNPs were identified with significant departure from HWE at significance level α<0.001 and were excluded in future analyses. To study the LD in the ancestral populations, pairwise linkage disequilibrium defined by D' values between SNPs on the same chromosome was calculated using the software Haploview in Whites. We identified 251 sets of adjacent SNPs in strong LD (95% lower bound D'>0.5). Since strong LD between adjacent markers can seriously bias the estimation of marker location-specific ancestry [26], we retained only one SNP from each of these sets in the subsequent analyses. After these data verification procedures, we defined an analytic set of 1,890 from the original 2,270 SNPs. These SNPs were entered in the hidden Markov model (HMM) to estimate the marker locus specific ancestry in African-Americans based on EM algorithm using software ADMIXPROGRAM [25].

### Estimation of marker location-specific ancestry

**Figure 4:**
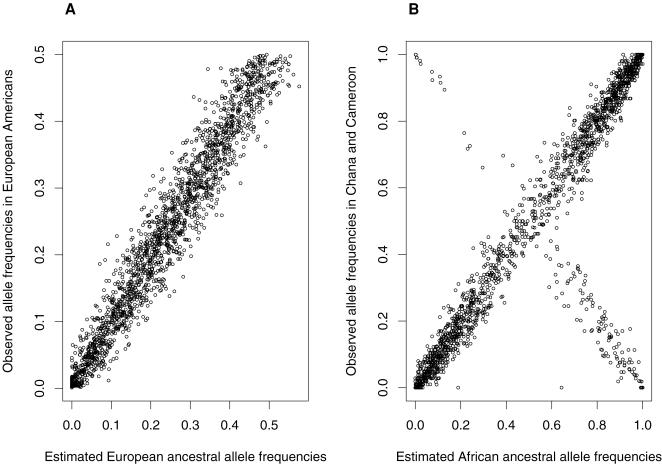
Ancestral allele frequencies. A. European ancestral allele frequencies estimated by Hidden Markov Model in the African-American sample vs the observed allele frequencies in European Americans. B. African ancestral allele frequencies estimated by Hidden Markov Model in the African-American sample vs the observed allele frequencies by the weighted average from Ghana and Cameroon obtained from Smith et al. (2004).

The genome-wide admixture mapping analysis was only performed in African-Americans since the SNP set of Smith et al. was specifically selected for the African-American population. We estimated marker location-specific ancestry using the HMM, in which the transmission probability was calculated based on the continuous gene flow (CGF) model [21], [25]. This method directly maximizes the likelihood function through an EM iterative algorithm and allows consideration of uncertainty of marker allele frequencies in the parental populations [25]. The number of generations since population admixture was estimated by maximizing the likelihood function for the number of generations. To estimate the marker location-specific ancestry, we assumed there were two parental populations for the African-American population. If our model fit the data well, we would expect the estimated allele frequencies in ancestral African and European populations to be close to what is observed in contemporary populations, although not the same. To allow for the uncertainty of ancestral marker allele frequencies, we compared the estimated ancestral allele frequencies and marker location-specific ancestries when no ancestral population information was used to a situation where only observed European allele frequencies were used as the initial European ancestral allele frequencies in the model. The results were essentially the same and we then reported the results using the European allele frequencies. The correlation between the observed and estimated allele frequencies in the European sample was 97.1% and the correlation between estimated African ancestral allele frequencies with that in panel described by Smith et al [31] (calculated from the weighted average allele frequencies of Ghana and Cameroon) was 84.5% (figure 4 A and B). The lower correlation for African allele frequencies is apparently due to inconsistent designation of the minor allele. However, this inconsistency has no effect to the results because no African ancestral information was used in our analysis.

### Testing for linkage by admixture mapping

To test for linkage we calculated two test statistics: the Z score of the case-only test and the Z score of the case-control test [21], [25]. We also estimated the marker location-specific ancestry using the software STRUCTURE [31], [50]. STRUCTURE was run under the linkage model without haplotype phase information, with 50,000 burn-in iterations followed by an additional 50,000 iterations. We specified SITEBYSITE = 1 and STRUCTURE provided output for the joint posterior assignment probabilities of population origin for the two alleles at each marker location. We next compared the marker-specific ancestry obtained with the software STRUCTURE to the results obtained from the EM method.

### Simulation of admixture populations

We directly simulated admixed populations according to the continuous gene flow model [21], [25]. The ancestral allele frequencies, the number of generations since the occurrence, and average ancestry were based on the estimates in the African American samples. To simulate African-American samples, at the first generation the marker genotypes of 10,000 unrelated people were simulated according to the African allele frequencies assuming HWE and independence of the markers. An admixed population was then formed by taking a proportion of 3.3% randomly selected from the simulated population to mate with people generated according to the marker allele frequencies in the European population, with the remaining individuals randomly mating among themselves. The number of children produced by each marriage was assumed to follow a Poisson distribution with mean size 2. The number of crossovers between two marker loci at a distance d cM was assumed to follow a Poisson distribution with mean d/100. This process was repeated in the following generations to form the current African American populations.

### Testing association between and marker and hypertension

We applied a logistic model regressing hypertension status on gender, age, age^2^ and the genotype effect assuming additive model. For African Americans, individual ancestry estimated by HMM was included in the model. For Mexican Americans, we applied the genome-control method [51] to control for population stratification by randomly selecting 100 unlinked SNPs. SNPs with significant association were further analyzed using different models of inheritance.

### Permutation test

To obtain the significance level of SNP rs2272996 in VNN1 accounting for multiple tests, we performed permutation tests for those SNPs with allele frequency difference between African Americans and Europeans greater than that of SNP rs2272996. We permutated the hypertension status together with gender, age, BMI and African ancestry 10,000 time and analyzed the individuals whose African ancestral>80% at each permutation. Logistic regression was then performed for each SNP and the minimum P-value was recorded at each permutation. These minimum P-values were tallied to obtain the null distribution of the test adjusting for multiple tests.

## Supporting Information

Table S1 Individual European Ancestral estimated by ADMIXPROGRAM and STRUCTURE
(0.14 MB XLS)

Table S2 Case-only and case-control Z-scores across the genome
(0.46 MB XLS)
